# Supplementary material for: Comparative genomics provides new insights into the diversity, physiology, and sexuality of the only industrially exploited tremellomycete: Phaffia rhodozyma
Source: BMC Genomics. 2016 Nov 9;17:901. doi: 10.1186/s12864-016-3244-7 (PMC5103461; doi:10.1186/s12864-016-3244-7)
Supplement: Additional file 6: — List of orphan genes with links to PFAM (related to Additional file 1: Table S1). (ZIP 1428 kb) [file 12864_2016_3244_MOESM6_ESM.zip › BLAST_HTML_FTR/G03444_P.html]

BLAST Search Results


```
BLASTP 2.2.27+


Reference:
Stephen F. Altschul, Thomas L. Madden, Alejandro A. Schäffer,
Jinghui Zhang, Zheng Zhang, Webb Miller, and David J. Lipman (1997),
"Gapped BLAST and PSI-BLAST: a new generation of protein database
search programs", Nucleic Acids Res. 25:3389-3402.


Reference for
composition-based statistics:
Alejandro A. Schäffer, L. Aravind, Thomas L. Madden, Sergei
Shavirin, John L. Spouge, Yuri I. Wolf, Eugene V. Koonin, and
Stephen F. Altschul (2001), "Improving the accuracy of PSI-BLAST
protein database searches with composition-based statistics and
other refinements", Nucleic Acids Res. 29:2994-3005.


Database: nr
           71,551,133 sequences; 26,053,659,533 total letters


Query= G03444_P

Length=973
                                                                      Score     E
Sequences producing significant alignments:                          (Bits)  Value

emb|CDZ96568.1|  hypothetical protein [Xanthophyllomyces dendrorh...  1986    0.0  
emb|CEM07891.1|  unnamed protein product [Vitrella brassicaformis...  51.6    0.004


 >emb|CDZ96568.1| hypothetical protein [Xanthophyllomyces dendrorhous]
Length=972

 Score = 1986 bits (5146),  Expect = 0.0, Method: Compositional matrix adjust.
 Identities = 972/972 (100%), Positives = 972/972 (100%), Gaps = 0/972 (0%)

Query  1    MTSRFIAHSSRRAPQNLALSAPSFHLSSGYTTRSFRRQSTFFHPWPSIKGKGVLRDLSDA  60
            MTSRFIAHSSRRAPQNLALSAPSFHLSSGYTTRSFRRQSTFFHPWPSIKGKGVLRDLSDA
Sbjct  1    MTSRFIAHSSRRAPQNLALSAPSFHLSSGYTTRSFRRQSTFFHPWPSIKGKGVLRDLSDA  60

Query  61   SGTYQILGARPFSTCLLSSRSPSRRNSRSVGLRHPVKNYRSSIDVSLFHPGVWEYELNDA  120
            SGTYQILGARPFSTCLLSSRSPSRRNSRSVGLRHPVKNYRSSIDVSLFHPGVWEYELNDA
Sbjct  61   SGTYQILGARPFSTCLLSSRSPSRRNSRSVGLRHPVKNYRSSIDVSLFHPGVWEYELNDA  120

Query  121  SRTTRSSSSGRKTQIDQSNSNPEHIPFFQPSPSSSSSPRSSTVWHPTPHLQHHTYIVPQE  180
            SRTTRSSSSGRKTQIDQSNSNPEHIPFFQPSPSSSSSPRSSTVWHPTPHLQHHTYIVPQE
Sbjct  121  SRTTRSSSSGRKTQIDQSNSNPEHIPFFQPSPSSSSSPRSSTVWHPTPHLQHHTYIVPQE  180

Query  181  IQPTYDPPMDAIEPPVSFRIQSILYHTSSPVAAFEPERIILPLLFSSSEPLDRLGIASED  240
            IQPTYDPPMDAIEPPVSFRIQSILYHTSSPVAAFEPERIILPLLFSSSEPLDRLGIASED
Sbjct  181  IQPTYDPPMDAIEPPVSFRIQSILYHTSSPVAAFEPERIILPLLFSSSEPLDRLGIASED  240

Query  241  FEQLVKCLVEQVKNENTELRDECARFVLRLVGQRPGLGSEVIGWAVQAALVLARKSQDFT  300
            FEQLVKCLVEQVKNENTELRDECARFVLRLVGQRPGLGSEVIGWAVQAALVLARKSQDFT
Sbjct  241  FEQLVKCLVEQVKNENTELRDECARFVLRLVGQRPGLGSEVIGWAVQAALVLARKSQDFT  300

Query  301  PVRELYQHLSPDASLALHAPFLLALLSARNRLLSIEAVERFLILTQKQKFLAHHLADPKS  360
            PVRELYQHLSPDASLALHAPFLLALLSARNRLLSIEAVERFLILTQKQKFLAHHLADPKS
Sbjct  301  PVRELYQHLSPDASLALHAPFLLALLSARNRLLSIEAVERFLILTQKQKFLAHHLADPKS  360

Query  361  YQRLFQKIFRHLATSREIEWMKKMIKRLDWLASQLKRHNSLCVAQQKMFSSIASFILTTC  420
            YQRLFQKIFRHLATSREIEWMKKMIKRLDWLASQLKRHNSLCVAQQKMFSSIASFILTTC
Sbjct  361  YQRLFQKIFRHLATSREIEWMKKMIKRLDWLASQLKRHNSLCVAQQKMFSSIASFILTTC  420

Query  421  DGHWSISSNSVFAARDDPSSSQLIDDSLSIYAMLISCQTIHLRSSSLVQFAFTFSSTRPA  480
            DGHWSISSNSVFAARDDPSSSQLIDDSLSIYAMLISCQTIHLRSSSLVQFAFTFSSTRPA
Sbjct  421  DGHWSISSNSVFAARDDPSSSQLIDDSLSIYAMLISCQTIHLRSSSLVQFAFTFSSTRPA  480

Query  481  CLVLTHPRINTRHFHLLAYKLIRSVDESGDEHTSFYGMRSFGQDLIEAIGKSVAATDRVI  540
            CLVLTHPRINTRHFHLLAYKLIRSVDESGDEHTSFYGMRSFGQDLIEAIGKSVAATDRVI
Sbjct  481  CLVLTHPRINTRHFHLLAYKLIRSVDESGDEHTSFYGMRSFGQDLIEAIGKSVAATDRVI  540

Query  541  SSDLLHTLLAFWIDLNQASEAAKLYLTIYRPSLTQSCTPAPPVHLSMILIRALLPSRTPV  600
            SSDLLHTLLAFWIDLNQASEAAKLYLTIYRPSLTQSCTPAPPVHLSMILIRALLPSRTPV
Sbjct  541  SSDLLHTLLAFWIDLNQASEAAKLYLTIYRPSLTQSCTPAPPVHLSMILIRALLPSRTPV  600

Query  601  CLDLAASIFDLIPMLDRTLELHNDLIRAGNLTFSTRSAANAFTDQIERSLNSHPKIKPDV  660
            CLDLAASIFDLIPMLDRTLELHNDLIRAGNLTFSTRSAANAFTDQIERSLNSHPKIKPDV
Sbjct  601  CLDLAASIFDLIPMLDRTLELHNDLIRAGNLTFSTRSAANAFTDQIERSLNSHPKIKPDV  660

Query  661  RTTEAFLSAPPMFYNPADPAERAVGRAEHALRDEIRSSLFENYFRLALNEGYAFRQETWA  720
            RTTEAFLSAPPMFYNPADPAERAVGRAEHALRDEIRSSLFENYFRLALNEGYAFRQETWA
Sbjct  661  RTTEAFLSAPPMFYNPADPAERAVGRAEHALRDEIRSSLFENYFRLALNEGYAFRQETWA  720

Query  721  LMIRAVGADVGPLEAEEVIDWYALHGGQVGKLIVNSLLKNEGTRRRRVGNSLMRSMMEVM  780
            LMIRAVGADVGPLEAEEVIDWYALHGGQVGKLIVNSLLKNEGTRRRRVGNSLMRSMMEVM
Sbjct  721  LMIRAVGADVGPLEAEEVIDWYALHGGQVGKLIVNSLLKNEGTRRRRVGNSLMRSMMEVM  780

Query  781  VKMKKSFPWFRPDWNTLTILIQVFVRWDADFPTGPFRRLFHQTMVLNQIPGASLFKSSFL  840
            VKMKKSFPWFRPDWNTLTILIQVFVRWDADFPTGPFRRLFHQTMVLNQIPGASLFKSSFL
Sbjct  781  VKMKKSFPWFRPDWNTLTILIQVFVRWDADFPTGPFRRLFHQTMVLNQIPGASLFKSSFL  840

Query  841  PVSTSSSSSSNFRSSSCSSSISANRFYTSASASFSTSISTLTSLSESTPASPIAASELTQ  900
            PVSTSSSSSSNFRSSSCSSSISANRFYTSASASFSTSISTLTSLSESTPASPIAASELTQ
Sbjct  841  PVSTSSSSSSNFRSSSCSSSISANRFYTSASASFSTSISTLTSLSESTPASPIAASELTQ  900

Query  901  RFPLDLDPDSTSLSLEEHIVPLYDLFIQGFEKREDYEAAEWAREGKRMARALEWEERRKR  960
            RFPLDLDPDSTSLSLEEHIVPLYDLFIQGFEKREDYEAAEWAREGKRMARALEWEERRKR
Sbjct  901  RFPLDLDPDSTSLSLEEHIVPLYDLFIQGFEKREDYEAAEWAREGKRMARALEWEERRKR  960

Query  961  RRTEGGEGRRSS  972
            RRTEGGEGRRSS
Sbjct  961  RRTEGGEGRRSS  972


>emb|CEM07891.1| unnamed protein product [Vitrella brassicaformis CCMP3155]
Length=672

 Score = 51.6 bits (122),  Expect = 0.004, Method: Compositional matrix adjust.
 Identities = 44/168 (26%), Positives = 82/168 (49%), Gaps = 28/168 (17%)

Query  236  IASEDFE----QLVKCLVEQVKNENTELRDECARFVLRLVGQRPGLGSEVIGWAVQAALV  291
            + S DF     +LV  L++++KNE   +R  C ++V  L+ +RPG          Q ALV
Sbjct  342  VNSSDFAFAVPELVTVLLDKLKNEGVTVRVACLQWVCMLLTRRPG----------QMALV  391

Query  292  LARKSQDFTPVRELYQHLSPDASLALHAPFLLALLS--------ARNRLLSIEAVERFLI  343
            L      F P+ E  +H  PD+ + + A  +LA +S           +LL + A +R + 
Sbjct  392  LEIL---FDPIFETLKH--PDSEVVVAALQVLAQISQHGDFFDLVSQKLLHLFAKDRKM-  445

Query  344  LTQKQKFLAHHLADPKSYQRLFQKIFRHLATSREIEWMKKMIKRLDWL  391
            L Q+ + +   L      Q+L+  I + L   +++E+  ++++  +W+
Sbjct  446  LDQRGRLVIRQLCTYLDVQQLYTTIAKALEQEQDLEYCHQLVQIFNWI  493


Lambda      K        H        a         alpha
   0.320    0.132    0.391    0.792     4.96 

Gapped
Lambda      K        H        a         alpha    sigma
   0.267   0.0410    0.140     1.90     42.6     43.6 

Effective search space used: 11801548821440


  Database: nr
    Posted date:  Sep 23, 2015 12:05 AM
  Number of letters in database: 26,053,659,533
  Number of sequences in database:  71,551,133


Matrix: BLOSUM62
Gap Penalties: Existence: 11, Extension: 1
Neighboring words threshold: 11
Window for multiple hits: 40
```
